# Comparison of *J* Integral Assessments for Cracked Plates and Pipes

**DOI:** 10.3390/ma14154324

**Published:** 2021-08-02

**Authors:** Ľubomír Gajdoš, Martin Šperl, Jan Bayer, Jiří Kuželka

**Affiliations:** 1 Institute of Theoretical and Applied Mechanics of the Czech Academy of Sciences, v. v. i. Prosecká 809/76, 190 00 Prague, Czech Republic; sperl@itam.cas.cz (M.Š.); bayer@itam.cas.cz (J.B.); 2 Faculty of Mechanical Engineering, Czech Technical University in Prague, Technická 4, 166 07 Prague, Czech Republic; jiri.kuzelka@fs.cvut.cz

**Keywords:** crack, stress intensity factor, *J* integral, stress concentration, strain energy density, Ramberg–Osgood relation, linepipe steels X52, X70

## Abstract

The purpose of this article is to compare two predictive methods of *J* integral assessments for center-cracked plates, single-edge cracked plates and double-edge cracked plates produced from X52 and X70 steels, and a longitudinally cracked pipe produced from X70 steel. The two methods examined are: the GSM method and the *J_s_* procedure of the French RCC-MR construction code, designated here as the FC method. The accuracy of *J* integral predictions by these methods is visualized by comparing the results obtained with the “reference” values calculated by the EPRI method. The main results showed that both methods yielded similar *J* integral values, although in most cases, the GSM predictions were slightly more conservative than the FC predictions. In comparison with the “reference” values of the *J* integral, both methods provided conservative results for most crack configurations, although the estimates for cracks of a relative length smaller than 1/8 were not found to be so conservative. The prediction of burst pressures for external longitudinal semielliptical part-through cracks in X70 steel pipe showed that the magnitudes of predicted burst pressures came very close to each other, and were conservative compared to FEM (finite element method) calculations and experimentally determined burst pressures.

## 1. Introduction

The aim of this study was to demonstrate that the newly modified GS method [1], now renamed as GSM method, provides reliable *J* integral predictions which compare well with predictions based on the generally accepted *J_s_* procedure of the French RCC-MR construction code [2]. Before defining the problems concerned with these predictions it should be stated that the *J* contour integral, as proposed by Rice [3], has gained recognition as a fracture-characterizing parameter in elastic–plastic solids. Being based on an energy balance approach, it can also be used as an elastic–plastic energy release rate (under certain restrictions). *J* integral is still used widely in several state-of-the-art papers, e.g., [4,5,6]. Generally, the determination of the *J* integral for a cracked body loaded with a certain stress pattern is not simple and the solution of this problem requires, with the exception of some simple bodies and crack configurations, employment of computerized numerical methods. In some situations, the exact determination of the *J* integral may not be required. What may be required (or at least desirable), however is a simple method that can show, from the viewpoint of integrity, whether the exploited structural component containing a crack-like defect is still safe to continue operating. In such cases, a conservative estimate of the *J* integral could provide essential information on the integrity of a cracked body. A more precise (and therefore expensive) FEM analysis would then only be used when this conservative approximate analytical method predicts failure. Therefore the relevancy of the study can be seen in that the developed GSM method provides a good conservative estimation of the *J* integral for various cracked components.

In contrast to *J* integral computation, determination of the stress intensity factor K, as a fracture characterizing the parameters of brittle materials, is much simpler. Several compendia on stress intensity factors exist, e.g., Tada et al. [7], Rooke and Cartwright [8], and Murakami [9], which enable various crack problems to be solved in the area of linear elastic fracture mechanics. From the definition of the *J* integral, it is clear that this quantity is dependent not only on the stress pattern, size, and configuration of a crack (like it is for K) but also on the stress–strain relationship of the material of the body. This consequently indicates that there is some relationship between the *J* integral, the stress intensity factor, and the stress–strain dependence. Although several approximate methods have been proposed for the determination of this relationship, we have concentrated on two of them: the GSM method and the FC method. The latter method, proposed as early as 1985 in Addendum A16 of the French nuclear code [2] as the *J_s_* method, became a subject of further development and was then integrated into the 2007 edition of the RCC-MR code as published by Marie et al. [10]. In this edition, the correction factor in the plastic zone *φ* (according to the denotation used in this article) has been altered compared to the 1985 edition. Further development of the AFCEN Codes yielded the RCC-MX Code in 2008, and finally the RCC-MRx Code in 2018. As reported by Muňoz Garcia et al. [11], the 2018 code contains a set of technical rules to be applied in the design of research reactors. It should be stated that the general formalism for the *J_s_* integral is based on the reference stress concept introduced by Ainsworth [12].

## 2. Background of the GSM and FC Methods

### 2.1. The GSM Method

This method is based on (i) a formal description of the *J* integral for a circular notch from the definition, (ii) substitution of the relative strain energy density along the periphery of the notch by a third power of the cosine function of the polar angle, and (iii) allowing the radius of the circular notch to go to zero in the final expression for the *J* integral. The idea of a formal description of the *J* integral, for a circular notch followed by reducing the notch radius to zero in the final expression for obtaining the *J* integral of a crack, is not new. There are some papers by Matvienko and Morozov [13,14] and by Matvienko [15] which demonstrate such an approach.

As shown by Norio and Yasuhiro [16], stress intensity factors can be determined from the limiting values of elastic stress concentration factors as the root radius *ρ* of the notch approaches zero. In the derivation of the *J* integral, the GSM method considers a symmetrically loaded notch with its tip embedded in a mode *I* stress field. The maximum stress *σ*_max_ occurs directly ahead of the notch. Dimensional considerations of the crack-tip stress field for an isotropic elastic body lead to:

(1) $$K_{I}{}^{p}=\frac{\sqrt{\pi }}{2}\;k_{t}\sigma_{n}\sqrt{\rho }$$

According to the definition, the *J* integral for a cracked body is given by the expression

(2) $$J=\int_{\Gamma }wdy-\int_{\Gamma }T_{i}\frac{\partial u_{i}}{\partial x}ds\;(i=1,2)$$

where

$w=\int_{0}^{\varepsilon_{ij}}\sigma_{ij}d\varepsilon_{ij}$ is the strain energy density
Γ is any contour encircling the tip of the crack in a counterclockwise direction
*T_i_* are the components of the traction vector
*u_i_* are the displacement vector components
*ds* is a length increment along the contour Γ

**Figure 1:**
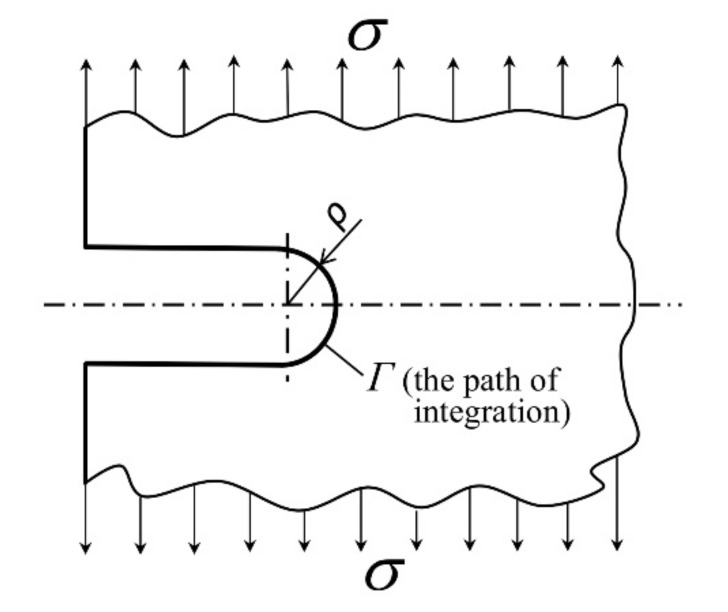
A section of a notched body, loaded by a remote uniform stress, and an indication of the path of integration.

Let us consider a body with external notches on both sides, loaded perpendicularly to the plane of the notches. A section of the notched body around the notch is shown in Figure 1.

**Figure 2:**
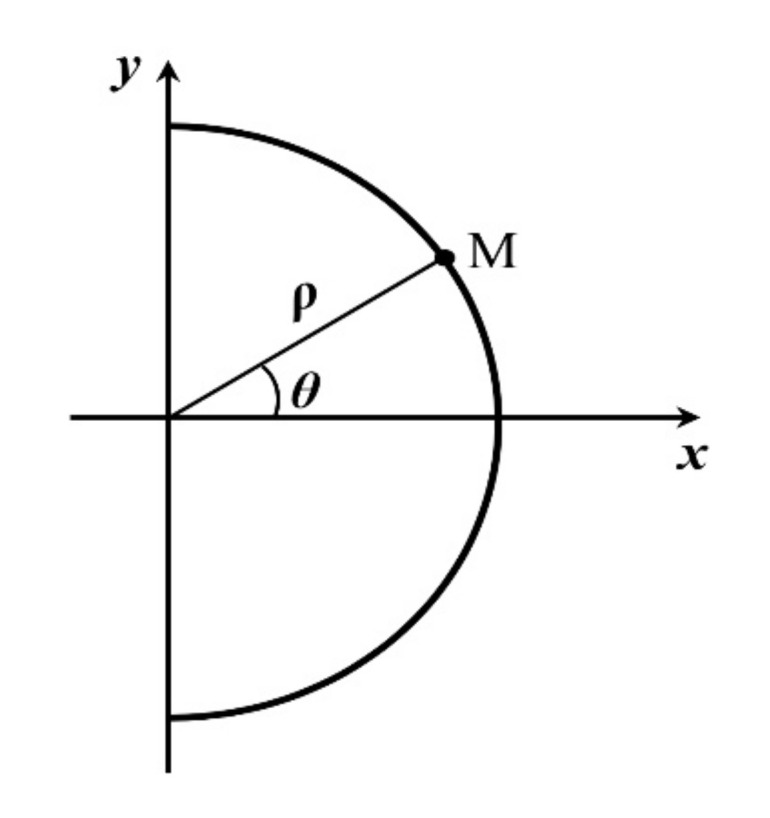
Polar co-ordinates of a point on the semicircular periphery of the notch.

The shape of the notch root is semicircular, the radius being *ρ*. By the concept of the invariance of the *J* integral, the value of the *J* integral does not depend on the path of integration. In its derivation the *J* integral is formally written for the notched body, and the path of integration is chosen so as to coincide with the periphery of the semicircular notch root (see Figure 1). Since the path of integration leads over a free surface, the second term on the right-hand side of Equation (2) becomes zero, so that after the transformation of Cartesian to polar co-ordinates of the points on the semicircular notch root (Figure 2), the *J* integral for the notched body takes the form:

(3) $$J=\int_{\Gamma }wdy=\int_{-\frac{\pi }{2}}^{+\frac{\pi }{2}}w_{\left(\theta \right)}\rho \cos\theta d\theta$$

The GSM method relates the strain energy density *w_(θ)_* at point M on the periphery of the notch root, characterized by the polar angle *θ*, to the maximum strain energy density *w*_max_ = *w_(θ=0)_*. The relation between *w_(θ)_* and *w*_max_ depends not only on the polar angle *θ* but also on the magnitude of the load. The results of finite element investigations into the strain energy density along a notch root in a double-edge notch panel [1] showed that the relative strain energy density (*w_(θ)_/w*_max_) can be substituted with a certain approximation by the function cos^3^*θ*. Considering this, Equation (3) can be rewritten as:

(4) $$J=\int_{\Gamma }wdy=\int_{-\frac{\pi }{2}}^{+\frac{\pi }{2}}w_{\max}\rho \cos^{4}\theta d\theta =\frac{3}{8}\pi \rho w_{\max}$$

The infinitesimal strain energy density *dw* is given in principal stresses and strains as:

(5) $$dw=\sigma_{1}d\varepsilon_{1}+\sigma_{2}d\varepsilon_{2}+\sigma_{3}d\varepsilon_{3}$$

In the notch root, characterized by *θ* = 0, the stress *σ*_2_ is always zero because of the free surface; the stress *σ*_3_ is zero for the plane stress state; and the strain *ε*_3_ is zero for the plane strain state. This means that the infinitesimal strain energy in the notch root (*θ* = 0) is reduced to

(6) $$dw=\sigma_{1}d\varepsilon_{1}$$

so that the strain energy density becomes

(7) $$w=\int dw=\int_{0}^{\varepsilon_{1}}\sigma_{1}d\varepsilon_{1}$$

or, with the notation used before:

(8) $$w=\int_{0}^{\varepsilon_{\max}}\sigma_{\max}d\varepsilon_{\max}$$

The GSM method supposes that material obeys the Ramberg–Osgood dependence (9) and that the hypothesis of equivalent strain energy density at the notch tip [17] can be applied:

(9) $$\frac{\varepsilon }{\varepsilon_{0}}=\frac{\sigma }{\sigma_{0}}+\alpha {\left(\frac{\sigma }{\sigma_{0}}\right)}^{n}$$

According to the concept of this hypothesis, the following equation holds

(10) $$w=w_{n}k_{t}{}^{2}$$

where *w_n_* is the energy density due to the net section stress *σ_n_*.

By combining (8) and (10), we arrive at:

(11) $$w=k_{t}{}^{2}\int_{0}^{\varepsilon_{n}}\sigma_{n}d\varepsilon_{n}.$$

By differentiating the Ramberg–Osgood relation (9), and considering that *ε_0_* = *σ_0/_E*, it is possible to arrive at:

(12) $$d\varepsilon =\left[\frac{1}{E}+\frac{\alpha n}{E}{\left(\frac{\sigma }{\sigma_{0}}\right)}^{n-1}\right]d\sigma$$

When applying this equation for *ε_n_* and *σ_n_* to Equation (11), the following expression for *w* is obtained:

(13) $$w=k_{t}{}^{2}\int_{0}^{\sigma_{n}}\sigma_{n}\left[\frac{1}{E}+\frac{\alpha n}{E}{\left(\frac{\sigma_{n}}{\sigma_{0}}\right)}^{n-1}\right]d\sigma_{n}=k_{t}{}^{2}\left[\frac{\sigma_{n}{}^{2}}{2E}+\frac{\alpha n\sigma_{0}{}^{2}}{\left(n+1\right)E}{\left(\frac{\sigma_{n}}{\sigma_{0}}\right)}^{n+1}\right]$$

By substituting *w*_max_ in Equation (4) with this expression, the *J* integral for a notch obtains the form:

(14) $$J=\frac{3}{8}\pi \rho k_{t}{}^{2}\left[\frac{\sigma_{n}{}^{2}}{2E}+\frac{\alpha n\sigma_{0}{}^{2}}{\left(n+1\right)E}{\left(\frac{\sigma_{n}}{\sigma_{0}}\right)}^{n+1}\right]$$

Recalling Equation (1), it is seen that the stress intensity factor for a crack can be expressed by:

(15) $$K_{I}=\underset{\rho \to 0}{\lim}\frac{k_{t}\sigma_{n}}{2}\sqrt{\pi \rho }$$

From there it follows as:

(16) $$\underset{\rho \to 0}{\lim}\rho k_{t}{}^{2}=\frac{4K^{2}}{\pi \sigma_{n}{}^{2}}$$

By combining Equations (14) and (15) we obtain:

(17) $$J=\frac{3}{4}\frac{K^{2}}{E}+\frac{3}{2}\frac{K^{2}}{E}\frac{\alpha n}{\left(n+1\right)}{\left(\frac{\sigma_{n}}{\sigma_{0}}\right)}^{n-1}$$

A multiple of four/three is applied to the first term in expression (17); this does not have a theoretical basis, but was incorporated to provide a well-known form of the elastic component of the *J* integral: *J_el_* = *K^2^/E′*, where *E′ = E* for plane stress and $E^{\prime }=E/\left(1-\nu^{2}\right)$ for plane strain, *ν* being Poisson´s number.

Equation (17) then obtains the form:

(18) $$J=\frac{K^{2}}{E^{\prime }}\left[1+\frac{3\alpha n}{2\left(n+1\right)}{\left(\frac{\sigma_{n}}{\sigma_{0}}\right)}^{n-1}\right]$$

Since the strain energy density *w(θ)* in Equation (4) was substituted with *w*_max_ cos^3^*θ* regardless of whether plane stress or plane strain conditions were concerned, the resulting Formula (18) can be used as a basis for the *J* integral assessment at conditions of plane stress and plane strain. As is known, the EPRI estimation scheme for the *J* integral [18] comes from stresses given by the HRR singularity and it arrives at the relationship simply expressed as:

(19) $$J_{pl}\approx {\left(P/P_{0}\right)}^{n+1}$$

*P_0_* can be defined arbitrarily, e.g., as the limit load *P_L_*. In order to make Equation (18) comply with this, a so-called limit load parameter *C*, by which the uniaxial yield stress *σ_0_* in (18) is to be multiplied, is introduced into the GSM method. The *C* parameter is given by Equation (20):

(20) $$C=\frac{P_{L}}{P}\frac{\sigma_{n}}{\sigma_{0}}$$

Equation (18) then obtains the form:

(21) $$J=\frac{K^{2}}{E^{\prime }}\left[1+\frac{3\alpha n}{2\left(n+1\right)}{\left(\frac{\sigma_{n}}{C\sigma_{0}}\right)}^{n-1}\right]$$

The limit load *P_L_* in Equation (20) can be determined as the product of the yield stress *σ_0_* and a certain geometrical function, which is specific for each panel and depends on the crack length *a* and the width *b* of a cracked panel of unit thickness. It is seen that the difference between the *J* assessment in the plane stress condition and in the plane strain condition is given (besides Young´s modulus *E′*) by the level of the limit load parameter *C*.

### 2.2. The FC Method

As already mentioned, the FC method was proposed in Addendum A16 of the French nuclear code [2] as the *J_s_* method, and its further development was published by Marie et al. [10]. The basis of this method was the R6 procedure [19], which made it possible to arrive at the following formula for the *J* calculation:

(22) $$J=J_{e}\left[\frac{\varepsilon_{ref}}{\varepsilon_{e}}+\varphi \right]$$

In Equation (22), *J_e_* is the elastic component of the *J* integral, *ε_ref_* is the reference strain corresponding to the reference stress *σ_ref_* defined by Equation (23), *ε_e_* = *σ_ref_*/*E* is the elastic strain, and *φ* is the plastic zone size-correction factor given in [10] by Equation (24):

(23) $$\sigma_{ref}=\sigma_{0}\frac{P}{P_{L}}$$

(24) $$\varphi =0.5\frac{{\left(\sigma_{ref}/\sigma_{0}\right)}^{2}}{{\left(\sigma_{ref}/\sigma_{0}\right)}^{2}+1}$$

It can be pointed out that, owing to Equation (20), the reference stress *σ_ref_* can also be written as:

(25) $$\sigma_{ref}=\frac{\sigma_{n}}{C}$$

**Figure 3:**
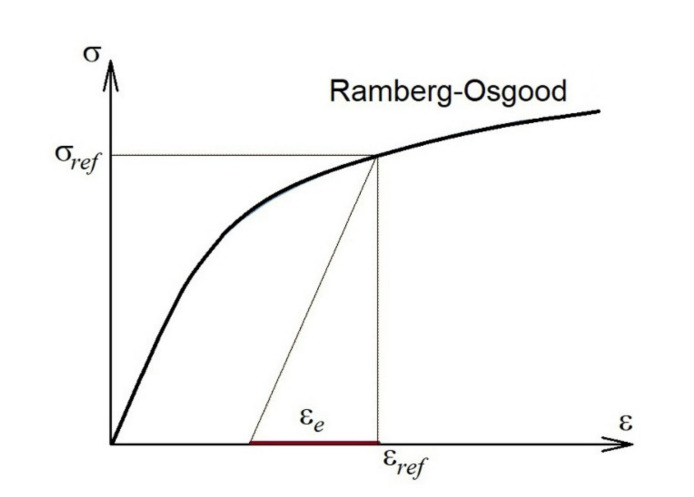
Ramberg–Osgood stress–strain diagram and denotation of the quantities used.

The first term in the brackets of Equation (22) reflects what experimentalists observed a long time ago, namely that at a certain load the *J* integral is proportional to the ratio of the actual strain to its elastic component. The quantities used in expression (22) are illustrated in Figure 3 for the Ramberg–Osgood approximation of the tensile curve of the material.

The Ramberg–Osgood dependence (9) can be rewritten by substituting *σ* with *σ_ref_* and *ε* with *ε_ref_* to obtain the form:

(26) $$\varepsilon_{ref}=\frac{\sigma_{ref}}{E}\left[1+\alpha {\left(\frac{\sigma_{ref}}{\sigma_{0}}\right)}^{n-1}\right]$$

By denoting

(27) $$1+\alpha {\left(\frac{\sigma_{ref}}{\sigma_{0}}\right)}^{n-1}=A$$

and considering $\frac{\sigma_{ref}}{E}=\varepsilon_{e}$

Equation (26) obtains the form:

(28) $$\varepsilon_{ref}=A\varepsilon_{e}$$

According to Equation (22), and considering Equations (24) and (28), the *J* integral is then expressed by Equation (29):

(29) $$J=J_{e}\left[A+0.5\frac{{\left(\sigma_{ref}/\sigma_{0}\right)}^{2}}{{\left(\sigma_{ref}/\sigma_{0}\right)}^{2}+1}\right]$$

This type of equation is also used in later editions of the RCC-MR code. The very last edition from 2018, denoting the RCC-MRx code, is not readily available from public sources. As follows from [11], it has been designed primarily for the mechanical components of high-temperature structures of nuclear installations; however, it can also be used for mechanical components of other types of nuclear installations. Although not mentioned explicitly in [11], it is likely that an equation of the type in (29) is also used in the RCC-MRx code, at least for some specific conditions like force-imposed mechanical loading, the modified limit load basis for the reference stress, and the Ramberg–Osgood description of the stress–strain curve.

Coming back to Equation (29), the fraction $\sigma_{ref}/\sigma_{0}$ in Equations (26) and (27) can be substituted, according to Equation (25), by $\sigma_{n}/\left(C\sigma_{0}\right)$ to obtain

(30) $$J=\frac{K^{2}}{E^{\prime }}\left[A+0.5\frac{{\left(\frac{\sigma_{n}}{C\sigma_{0}}\right)}^{2}}{\left[{\left(\frac{\sigma_{n}}{C\sigma_{0}}\right)}^{2}+1\right]}\right]$$

where

(31) $$A=1+\alpha {\left(\frac{\sigma_{n}}{C\sigma_{0}}\right)}^{n-1}$$

## 3. Use of the GSM and FC Methods for Cracked Plates

### 3.1. Description of Procedure

Before beginning to describe the procedure of comparing predictions using the GSM and FC methods, a few notes regarding the main way in which the GS method was modified, should be considered. It is worth noting that the first modification was made a few years ago when the so-called plastic constraint factor on yielding C′ was introduced to account for crack-tip constraint in cracked pipes from pipeline steels. On the basis of experimentally determined fracture pressures for part-through axial cracks of known dimensions, the C′ factor was found to vary between 2.0 and 2.4 for relative crack depths *a/t* ranging between 0.57 and 0.72 [20,21,22]. This enabled us to predict critical conditions for pipes containing deep part-through cracks. Owing to the limited group of steels tested and the narrow range of crack dimensions, there was a need for a more general approach to determine the *J* integral for various types of components. This is why the limit load concept was used in the GSM method instead.

For verification of the Formulas (21) and (30), it is necessary to compare the results calculated on the basis of these formulae with those obtained by exact calculations, mostly using finite-element analysis. The verification should be done for a wide range of crack sizes, component geometries, and loadings. However, in general, this requires versatile elastic–plastic computer programs. On the other hand, several simplified post-yield fracture mechanics methods, even if based on finite elements calculations, have been developed. Among these, the EPRI method [18] seems to be very convenient for the verification of the formulae derived, although it has been demonstrated in some papers that the EPRI method contains errors and inaccuracies in some of the *J* estimates. However, the EPRI method is easy to apply for simple component geometries and it utilizes the Ramberg–Osgood description of the stress–strain relationship of the material. It is widely used, so there is no need to describe it here.

**Figure 4:**
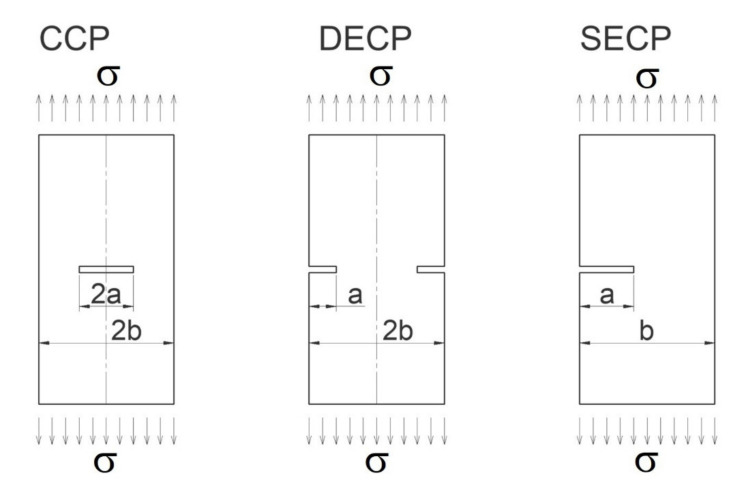
Specimens used in the investigations.

With a view to test specimen configurations for which the EPRI method offers *J* integral solutions, the following specimens have been considered: (i) center-cracked panel (CCP), (ii) double-edge cracked panel (DECP), and (iii) single-edge cracked panel (SECP). These panels are shown schematically in Figure 4.

**Figure 5:**
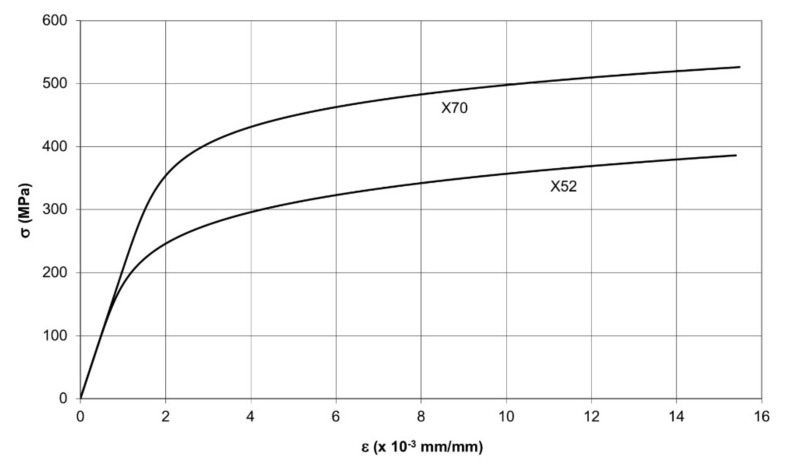
The stress-strain curves for the steels used.

Since the plastic component of the *J* integral strongly depends on the strain hardening exponent *n*, it is advisable to consider materials with various *n* values. For this reason, two pipeline steels were taken into account: X52 and X70. These steels were manufactured by Mannesmann company, Siegen, Germany. The stress–strain curves for these steels, as approximated by the Ramberg–Osgood relationship, are shown in Figure 5. The Ramberg–Osgood parameters, as well as the magnitudes of the U.T.S. for these steels are presented in Table 1.

The procedure for the verification of the GSM and FC methods consists in several steps. Firstly, it is necessary to determine the limit load parameter *C* according to Equation (20) for each type of specimen (panel) in both the plane stress and plane strain condition. The limit loads for the specimens used in the investigation can readily be found in various publications, e.g., [23,24]. They are recapitulated here:

CCP specimen

$\begin{array}{ll} P_{L}=2\left(b-a\right)\sigma_{0} & plane\;stress\\ P_{L}=\frac{4}{\sqrt{3}}\left(b-a\right)\sigma_{0} & plane\;strain \end{array}$

DECP specimen

$\begin{array}{ll} P_{L}=\frac{4}{\sqrt{3}}\left(b-a\right)\sigma_{0} & plane\;stress\\ P_{L}=b\left[0.72+1.82\left(1-\frac{a}{b}\right)\right]\sigma_{0} & plane\;strain \end{array}$

SECP specimen

$\begin{array}{ll} P_{L}=1.072\psi \left(b-a\right)\sigma_{0} & plane\;stress\\ P_{L}=1.455\psi \left(b-a\right)\sigma_{0} & plane\;strain \end{array}$ where

(32) $$\psi =\sqrt{1+{\left(\frac{a/b}{1-a/b}\right)}^{2}}-\frac{a/b}{1-a/b}$$

The results of transforming these limit loads to the limit load parameters *C* for CCP, DECP, and SECP specimens are shown in Table 2.

In the next step, the stress-intensity factors *K* are determined. For this purpose, the handbook [7] cited earlier is used. For the specimens used, the formulae for the *K* determination have the common form

(33) $$K=f\left(a/b\right)\sigma \sqrt{\pi a}$$

where *σ* is gross section stress and the function $f\left(a/b\right)$ is specific for each type of specimen: i.e., *f_CCP_* for CCP specimens, *f_DECP_* for DECP specimens, and *f_SECP_* for SECP specimens. The mathematical notations for these functions are the following:

(34) $$f_{CCP}=\frac{1-0.5\left(a/b\right)+0.37{\left(a/b\right)}^{2}-0.044{\left(a/b\right)}^{3}}{\sqrt{1-a/b}}$$

(35) $$f_{DECP}=\frac{1.122-0.56\left(a/b\right)-0.205{\left(a/b\right)}^{2}+0.471{\left(a/b\right)}^{3}-0.19{\left(a/b\right)}^{4}}{\sqrt{1-a/b}}$$

(36) $$f_{SECP}=\frac{0.752+2.02\left(a/b\right)+0.37{\left(1-\sin\frac{\pi a}{2b}\right)}^{3}}{\cos\frac{\pi a}{2b}}\sqrt{\frac{tg\frac{\pi a}{2b}}{\frac{\pi a}{2b}}}$$

Knowing the magnitudes of the limit load parameter C and the magnitudes of the stress-intensity factor *K*, the *J* integrals can be calculated by the GSM and FC methods using Equations (21) and (30) with (31). As it is more convenient in the comparison of the two investigated methods to use gross section stress *σ* instead of the net section stress *σ_n_*, it is necessary to transform *σ_n_* to *σ* in Equations (21), (30) and (31).

### 3.2. Results of Calculations

#### 3.2.1. The Center-Cracked Panel (CCP)

**Figure 6:**
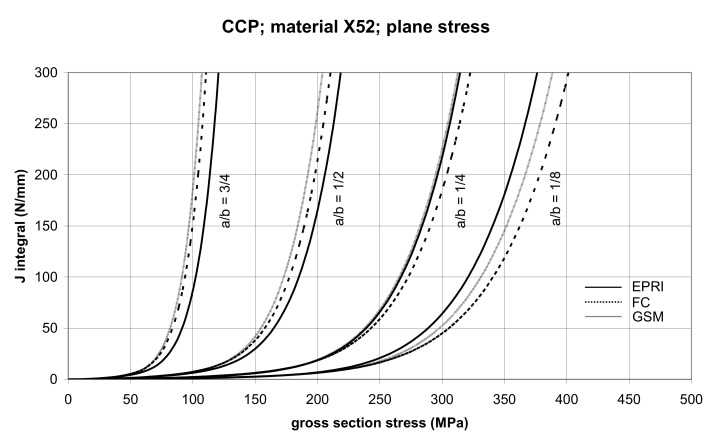
Comparison of *J* integral for CCP X52 steel specimens in the plane stress condition as determined by the EPRI, GSM, and FC methods.

**Figure 7:**
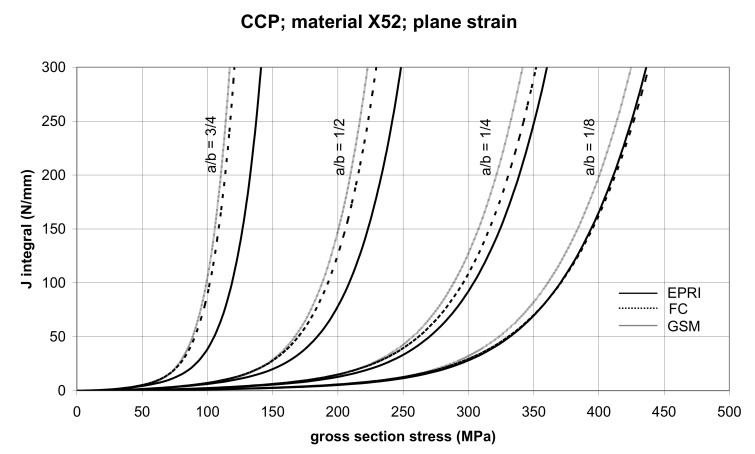
Comparison of *J* integral for CCP X52 steel specimens in the plane strain condition as determined by the EPRI, GSM, and FC methods.

**Figure 8:**
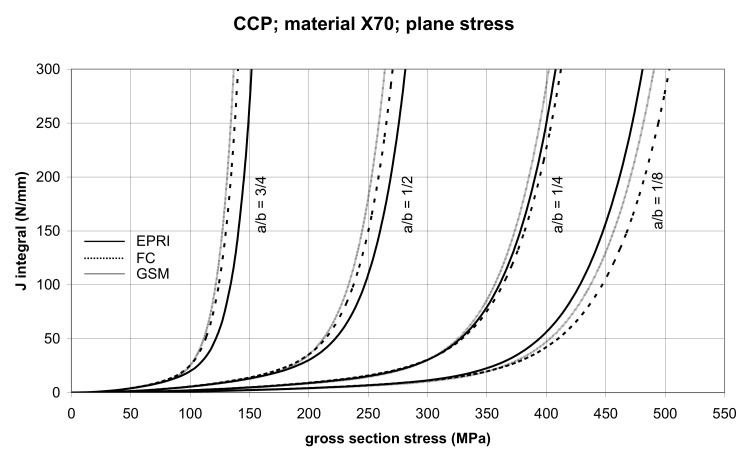
Comparison of *J* integral for CCP X70 steel specimens in the plane stress condition as determined by the EPRI, GSM, and FC methods.

**Figure 9:**
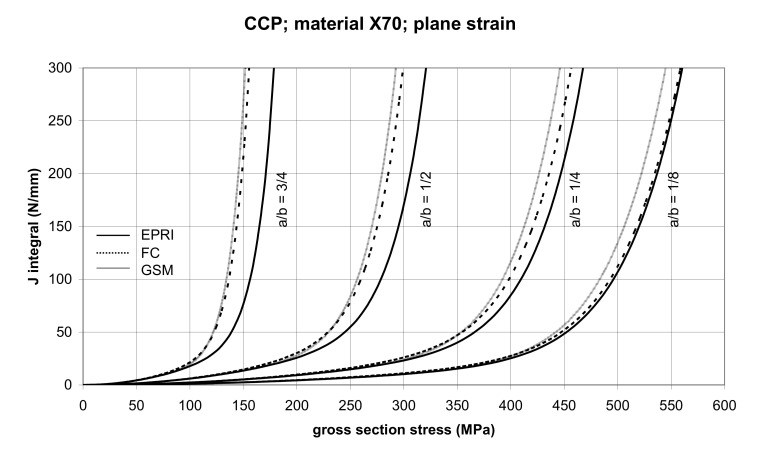
Comparison of *J* integral for CCP X70 steel specimens in the plane strain condition as determined by the EPRI, GSM, and FC methods.

The comparisons of *J* values, determined using the GSM and FC methods with the EPRI procedure, are presented in Figure 6 and Figure 7 for specimens made from X52 steel, and in Figure 8 and Figure 9 for specimens made from X70 steel.

#### 3.2.2. The Double-Edge Cracked Panel (DECP)

**Figure 10:**
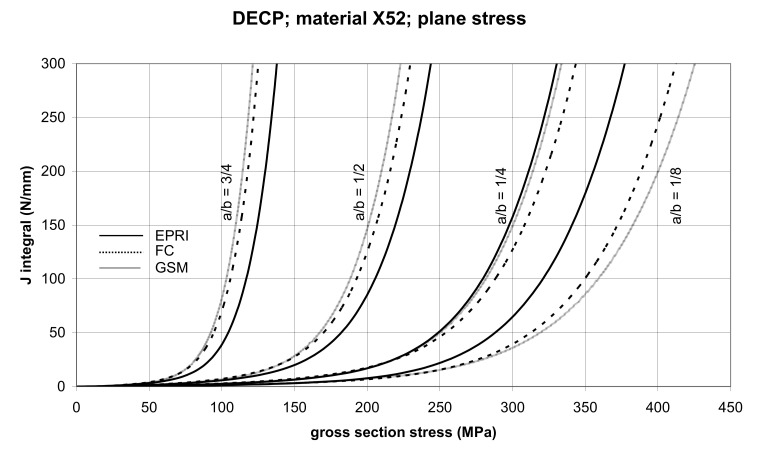
Comparison of *J* integral for DECP X52 steel specimens in the plane stress condition as determined by the EPRI, GSM, and FC methods.

**Figure 11:**
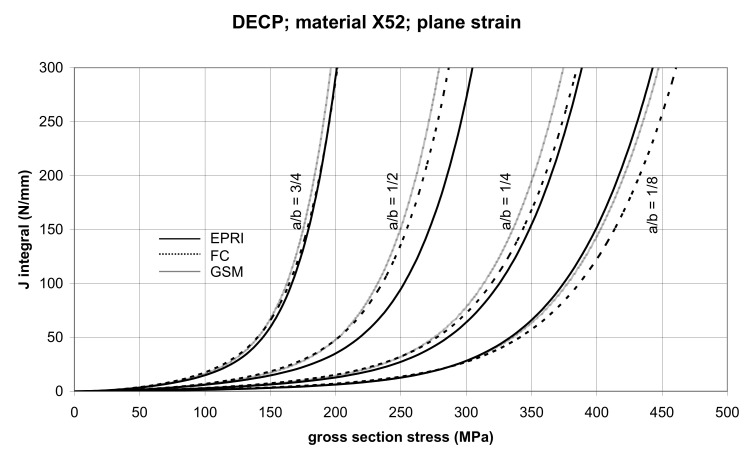
Comparison of *J* integral for DECP X52 steel specimens in the plane strain condition as determined by the EPRI, GSM, and FC methods.

**Figure 12:**
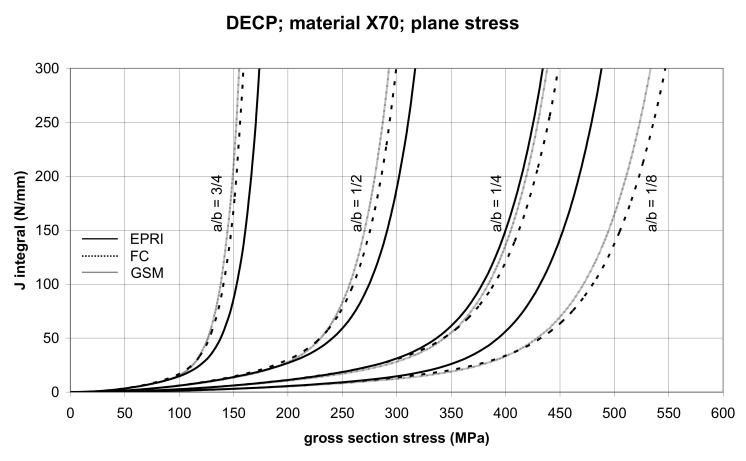
Comparison of *J* integral for DECP X70 steel specimens in the plane stress condition as determined by the EPRI, GSM, and FC methods.

**Figure 13:**
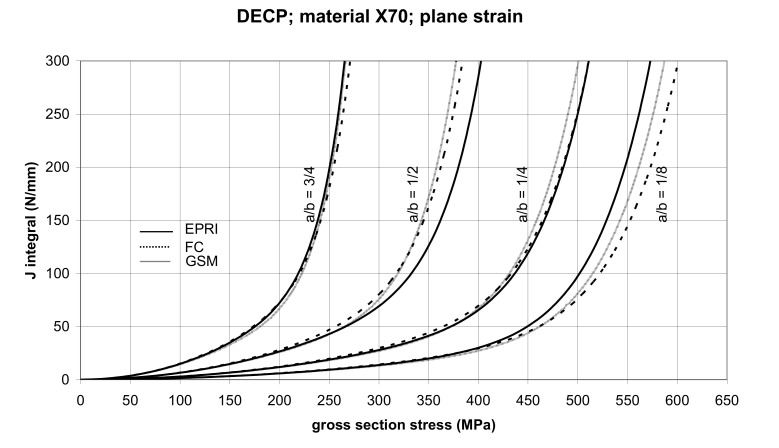
Comparison of *J* integral for DECP X70 steel specimens in the plane strain condition as determined by the EPRI, GSM, and FC methods.

The comparison of *J* values, determined by the GSM and FC methods, with the EPRI procedure is presented in Figure 10 and Figure 11 for specimens made from X52 steel, and in Figure 12 and Figure 13 for specimens made from X70 steel.

#### 3.2.3. The Single-Edge Cracked Panel (SECP)

**Figure 14:**
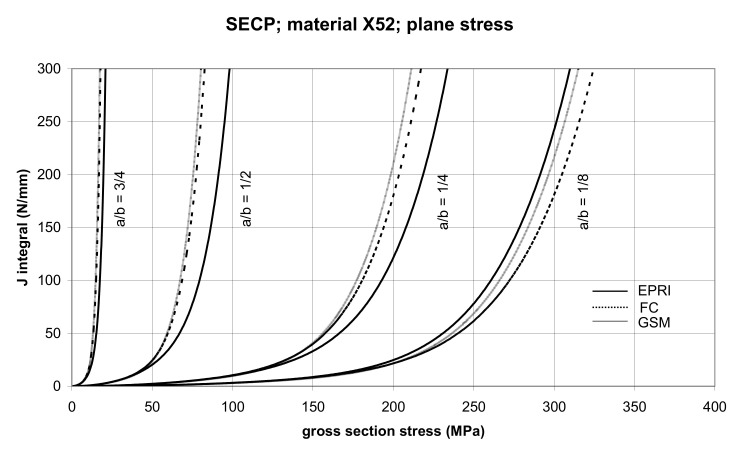
Comparison of *J* integral for SECP X52 steel specimens in the plane stress condition as determined by the EPRI, GSM, and FC method.

**Figure 15:**
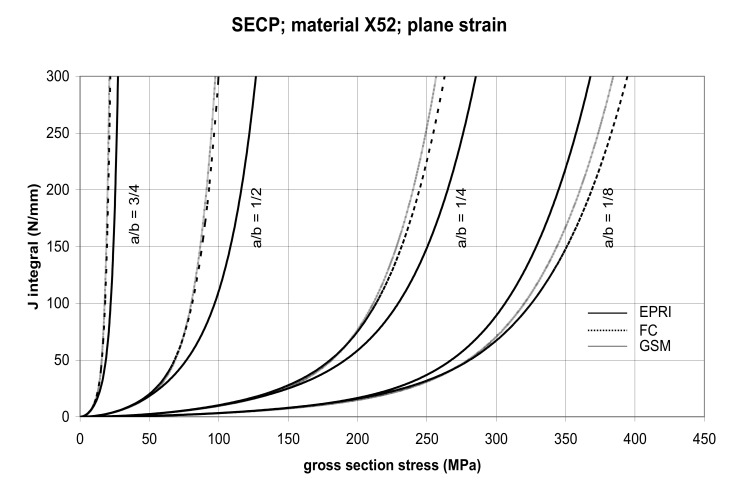
Comparison of *J* integral for SECP X52 steel specimens in the plane strain condition as determined by the EPRI, GSM, and FC methods.

**Figure 16:**
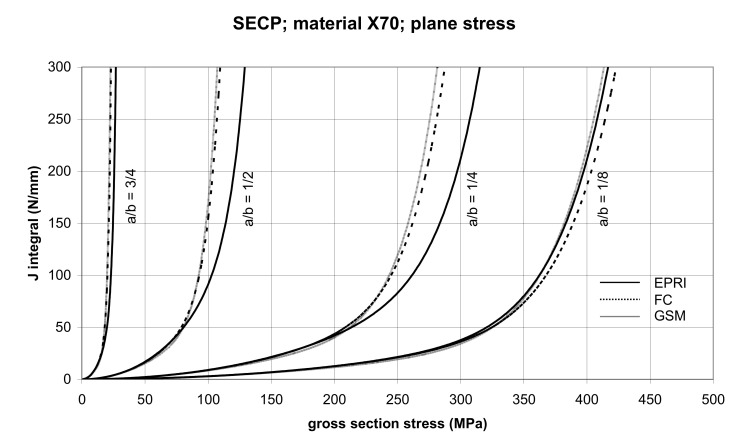
Comparison of *J* integral for SECP X70 steel specimens in the plane stress condition as determined by the EPRI, GSM, and FC methods.

**Figure 17:**
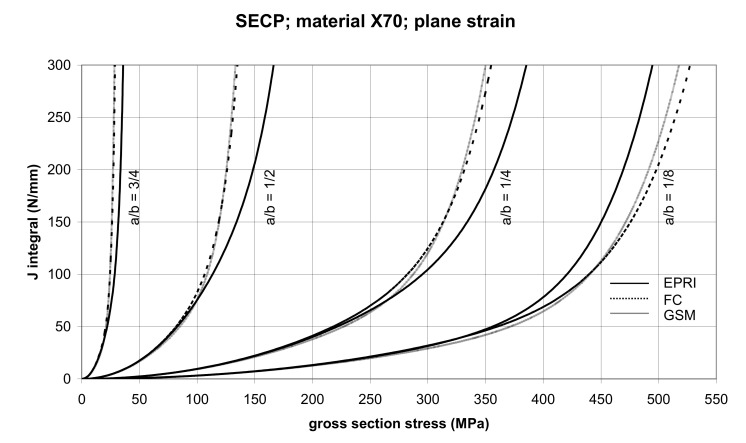
Comparison of *J* integral for SECP X70 steel specimens in the plane strain condition as determined by the EPRI, GSM, and FC methods.

The comparison of *J* values, determined by the GSM and FC methods, with the EPRI procedure is presented in Figure 14 and Figure 15 for specimens made from X52 steel, and in Figure 16 and Figure 17 for specimens made from X70 steel.

## 4. Use of the FC and GSM Methods for Cracked Pipes

### 4.1. Preparation of Pipe Segment

The verification of simplified engineering methods is best when conducted on real cracked components. Simplified methods, such as the FC and GSM methods, can be used, e.g., in the assessment of the integrity of pressure gas pipelines. They can be damaged by corrosion defects at the outside surface when corrosion protection fails [25]. The technical state of gas pipelines is therefore periodically assessed. This becomes increasingly significant when the planned lifetime of gas pipelines is close to expiring [26]. It is then necessary to increase the frequency of inspections of pipelines to identify in time the most dangerous of defects in pipelines—cracks. In order to evaluate critical conditions for a crack (crack size, gas pressure), it is very important to assess the *J* integral as the fracture-characterizing parameter and compare it with the fracture toughness *J_m_* of the pipe material.

For this purpose, we conducted tests on a segment taken from a gas pipeline made from X70 steel, measuring 1018 mm in outside diameter and 11.7 mm in wall thickness. The effective length of the pipe segment (the distance between the welds in dished bottoms) was approximately 3.5 *D*, where *D* was the outside diameter. A ring approximately 300 mm long was also cut from the pipeline to manufacture specimens for testing the mechanical and fracture-mechanical properties of the pipe material.

The tensile specimens were orientated circumferentially, and orientation of the CT specimens was such that crack-starter notches were axial. A curved semiproduct from the ring was press-straightened and then used to manufacture flat specimens for tensile tests. The tensile properties, namely the yield stress *σ_Y_* = *σ_0_* and *U.T.S.* = *R_m_* (including Ramberg–Osgood parameters determined subsequently), are presented in Table 1 for X70 steel. The fracture toughness of the steel was determined on the basis of the *J* integral with the *J_m_* parameter used as fracture toughness. The magnitude of the *J_m_* parameter was found to be 439 N/mm.

### 4.2. Procedure of the Tests and Experimental Results

Two types of part-through longitudinal slits were cut on the outside surface of the pipe segment; two working slits and a check slit. The check slit was approximately the same surface length as the working slits, but its depth was greater. Because the pipe segment was cycled by internal water pressure in order to initiate and develop a fatigue crack, the check slit functioned as a safety measure to prevent cracks that developed at the working slits from penetrating through the pipe wall. Efforts were made in the fracture tests to keep the hoop stress below the yield stress, because the operating stress in gas pipelines is around one half of the yield stress (and at present it does not exceed two thirds of the yield stress even in intrastate high-pressure gas transmission pipelines). Calculations revealed that in order to ensure the fracture pressure be less than the yield pressure, the depth of axial semi-elliptical cracks should be greater than one half of the wall thickness. If the crack depth was to have a certain magnitude before the fracture test began, the depth of the starting slit should be smaller than this magnitude by the fatigue extension of the crack along the perimeter of the slit tip. At the same time, we should bear in mind that the higher the fatigue extension of the crack, the better the agreement with the real crack.

**Figure 18:**
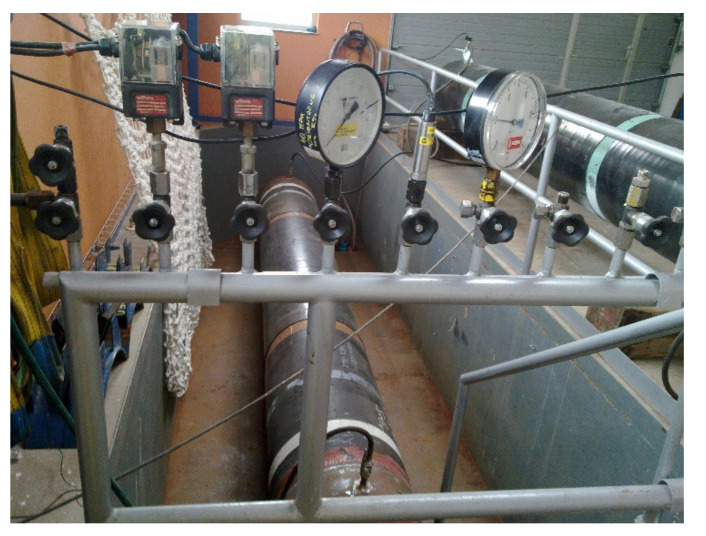
A view of testing pipes in the working pit.

In cycling the cracks, the water pressure fluctuated between *p*_min_ = 1.5 MPa and *p*_max_ = 5.3 MPa, and the number of pressure cycles was between 3000 and 4000. The period of a cycle was approximately 150 s. The cycling went on until a crack, initiated in the check slit, became a through crack. This moment was easy to detect because it was accompanied by a water leak. In order to run a test on a fracture it was necessary to remove the check slit, which had penetrated through the wall of the test segment, and to repair the wall, e.g., by welding a patch on it. Afterward, the pipe segment was loaded by increasing water pressure until it burst. Testing of the pipe is shown in the photograph (Figure 18).

After the first burst test was performed, the damaged part of the jacket were cut out and replaced by a patch welded in instead. A second burst test then followed. Afterward, the exact magnitudes of the surface half-crack length *c*, the crack depth at fracture *a_f_*, and the fracture pressure *p_f_*, were determined for both cracks, denoted by the letters A and B. They are presented in Table 3. The flow stress *σ_fs_* in Table 3 is considered as 1.1 × *σ_0_*.

### 4.3. Verification of Applicability of FC and GSM Method for Pipes

For verification of the fracture conditions for cracks A and B in the pipe segment, as predicted by the FC and GSM methods, we determined the fracture pressure *p_f_* for both cracks and compared the results with the FE prediction and experiment. Due to the fact that in the FC and GSM methods the *J* integral is determined as a function of (i) crack dimensions, (ii) crack plane section stress, and (iii) the stress–strain properties of the material, the fracture pressure was determined based on the condition that *J* integral is equal to its critical value—the fracture toughness.

**Figure 19:**
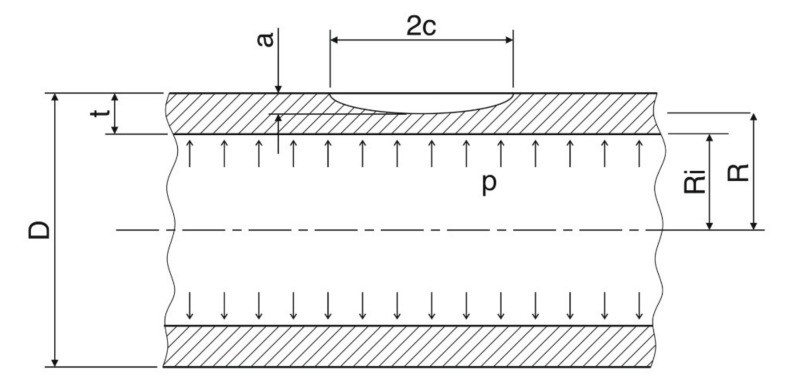
A longitudinal semi-elliptical part-through crack in a thin-walled pipe.

In principle, we proceeded in the same way as we did in Chapter 3 for CCP, DECP, and SECP specimens. This means that we firstly determined the limit load parameter *C*, and then modified the basic equations of the FC and GSM methods for a thin-walled cylindrical shell with a longitudinal semi-elliptical part-through crack as illustrated in Figure 19.

To be consistent with the form of the *J* integral, relations for the CCP, DECP, and SECP panels, we started with Equation (20). Considering $P/P_{L}=p/p_{L}$, where *P* is the load-per-unit length acting on the pipe wall in the circumferential direction due to pressure *p*, and *P_L_* is the limit load due to the limit pressure *p_L_*, Equation (20) obtains the form:

(37) $$C=\frac{\sigma_{n}}{\sigma_{0}}\frac{p_{L}}{p}=\frac{p_{L}}{p_{Y}}$$

Referring to the R6 method [27], we assumed that the hoop stress *σ_n_* could be written as

(38) $$\sigma_{n}=\frac{\sigma_{\varphi }}{\eta }$$

where

(39) $$\eta =1-\frac{\pi ac}{2t\left(2c+t\right)}$$

Equation (39) is considered satisfactory for 0.1 < *a*/(2*c*) < 0.5 and *a*/*t* ≤ 0.8. For *a*/(2*c*) < 0.1 the parameter *η* is equal to (1 − *a*/*t*). The yield pressure *p_Y_* can be determined by inserting *σ_n_ = σ_0_* into (38), and expressing *σ_φ_* as *p_Y_ R/t*. We then obtained:

(40) $$p_{Y}=\frac{\sigma_{0}t\eta }{R}$$

There are several formulae for the determination of the limit pressure *p_L_* for a longitudinal semi-elliptical part-through crack in a thin-walled pipe. We can use the Formula (41) which is published in [23]

(41) $$p_{L}=\sigma_{fs}\frac{t}{R_{i}}\xi$$

where

(42) $$\xi =1-\frac{a}{t}+\frac{a/t}{\sqrt{1+1.61c^{2}/\left(R_{i}a\right)}}$$

The symbol *σ_fs_* in (41) stands for the flow stress, taken as 1.1 times the yield stress σ_0_. The symbol *R_i_* in (41) and (42) stands for the internal radius, as illustrated in Figure 19. As stated in [23], Equations (41) and (42) give a lower bound estimate of the global collapse pressure. Owing to this, fracture pressures predicted by the GSM and FC methods will be more conservative.

After substituting *p_L_* in (37) by (41) and *p_Y_* in (37) by (40), and considering *σ_fs_* = 1.1 × *σ_0_* we arrived at (43):

(43) $$C=1.1\frac{R}{R_{i}}\frac{\xi }{\eta }$$

It can be seen from this equation that the limit load parameter C depended on (i) the ratio of the mean radius to the internal radius, and (ii) the geometrical parameters of the pipe and the crack. In the next step we determined the stress intensity factor K for the configuration displayed in Figure 19. We started with the stress intensity factor for a semi-elliptical part-through crack in a sheet. It was found that, for this case, a good engineering assessment of the stress intensity factor was provided by Newman [28]. A modified form of his solution for a longitudinal semi-elliptical part-through crack in a thin-walled pipe is expressed by the relation (44)

(44) $$K_{I}=\left[M_{F}+\left(E_{k}\sqrt{c/a}-M_{F}\right){\left(\frac{a}{t}\right)}^{s}\right]\frac{\sigma_{\varphi }\sqrt{\pi a}}{E_{k}}M_{TM}$$

where *M_F_* is a function dependent on the geometry of a crack (ratio *a*/*c*), $E_{k}=\int_{0}^{\pi /2}\sqrt{1-\frac{c^{2}-a^{2}}{c^{2}}\sin^{2}\theta d\theta }$ is an elliptical integral of the second kind, *s* is a function dependent on the geometry of a crack (ratio *a*/*c*) and on its relative depth *a*/*t* and, $M_{TM}=\frac{\left(1-\frac{a/t}{M_{T}}\right)}{\left(1-a/t\right)}$ is the correction factor for the curvature of the cylindrical shell and for an increase in stress owing to radial strains in the vicinity of the crack tip.

In the last relationship, *M_T_* is the Folias correction factor determined by the relation (45):

(45) $$M_{T}=\sqrt{1+1.255\frac{c^{2}}{Rt}-0.0135\frac{c^{4}}{R^{2}t^{2}}}$$

In order to compare the conservatism of *J* predictions made by the GSM and FC methods, we used the two methods to construct *J*–*p* dependences for longitudinal part-through cracks with the surface half-length *c* = 5 *t* and the relative depth *a/t* = 1/3, 1/2, and 3/4 in the X70 steel pipe segment. To do so we first modified Equations (21), (30) and (31) to obtain

(46) $$J=\frac{K^{2}}{E^{\prime }}\left[1+\frac{3\alpha n}{2\left(n+1\right)}{\left(\frac{pR_{i}}{1.1\xi t\sigma_{0}}\right)}^{n-1}\right]$$

(47) $$J=\frac{K^{2}}{E^{\prime }}\left[A+0.5\frac{{\left(\frac{pR_{i}}{1.1\xi t\sigma_{0}}\right)}^{2}}{\left[{\left(\frac{pR_{i}}{1.1\xi t\sigma_{0}}\right)}^{2}+1\right]}\right]$$

(48) $$A=1+\alpha {\left(\frac{pR_{i}}{1.1\xi t\sigma_{0}}\right)}^{n-1}$$

where *ξ* is given by the relation (42).

**Figure 20:**
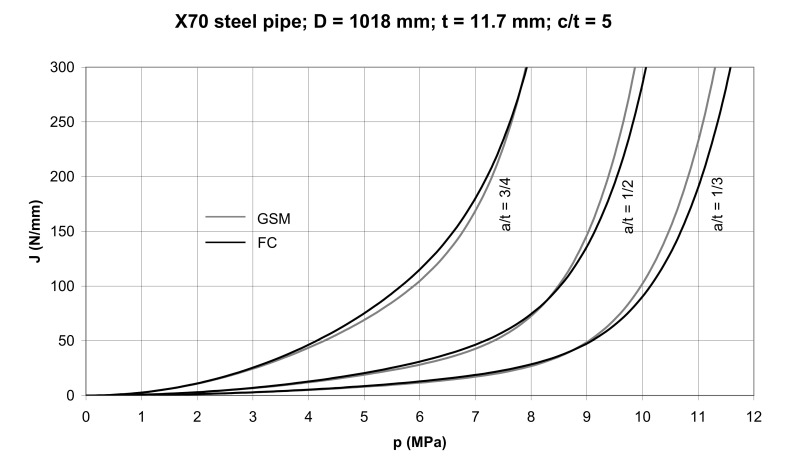
The *J*–*p* dependences for the X70 steel pipe segment with part-through cracks of various relative depths and of relative surface length *2c/t* = 10.

The *J–p* dependences, determined by Equations (46)–(48), are presented in Figure 20 for the X70 steel pipe segment. The parameters *α*, *n*, *σ_fs_*, as used in Equations (46)–(48), are those given in Table 3.

**Figure 21:**
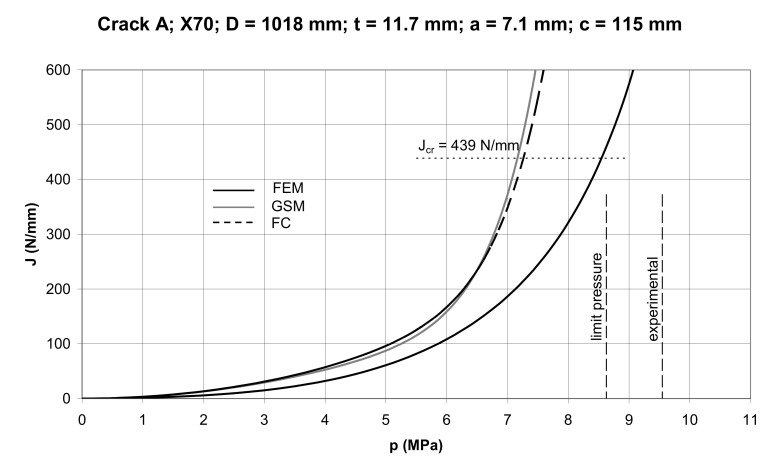
*J–p* dependences for Crack A.

**Figure 22:**
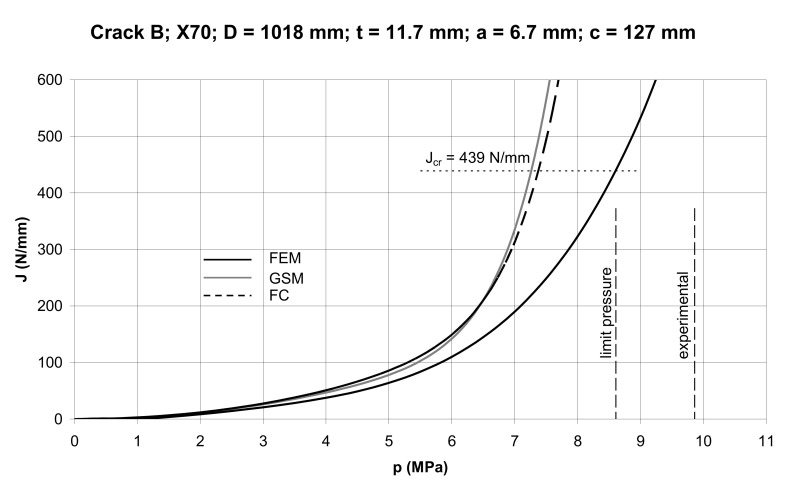
*J–p* dependences for Crack B.

As can be seen in Figure 20, the GSM method is more conservative than the FC method; the difference in the conservatism of the *J* prediction decreasing with the crack depth. It becomes practically negligible for the relative crack depth *a/t* = 3/4. By substituting concrete sizes for crack A and B into Equations (46)–(48) we can construct *J*–*p* dependences for these cracks using the GSM and FC methods as illustrated in Figure 21 and Figure 22. These curves are compared to the FEM curves computed using the ABAQUS software (ABAQUS Deutschland GmbH, München, Germany). The quarter pipe model with a longitudinal external crack was created. The pipe was loaded by internal pressure and corresponding axial stress. The magnitudes of the internal pressure ranged from 0–9 MPa with a step of 0.5 MPa. The FE-based commercial software ABAQUS was used for the evaluation of the *J* integral around the crack front in the plane of symmetry. In the region of interest, the elements with full integration and hybrid formulation were used. The characteristic element length at the vicinity of the crack was about 0.05mm.

It is seen here that the fracture pressure is determined as an *x* co-ordinate of the point of intersection of the appropriate curve with the horizontal *J_cr_*. The limit pressure given by Equation (41) with Equation (42), and the fracture pressure determined experimentally, are also represented here.

Data regarding the geometry of the pipe, the sizes of cracks at fracture, as well as the actual fracture pressures, are summarized in Table 4. The results of determining the fracture pressures using the GSM, FC, and FE methods (*p*_GS_, *p*_FC_, *p*_FE_) from the diagrams in Figure 21 and Figure 22, limit pressures p_L_ determined by Equation (41), and experimental pressures *p*_f_, are presented in Table 5.

## 5. Discussion of Results

### 5.1. Cracked Panels

Before evaluating and discussing the *J* integral predictions made using the FC and GSM methods, it should be noted that the resulting *J–σ* curves were compared to the “reference” *J–σ* curves determined by the EPRI method. With some exceptions, the EPRI curves can be considered to be sufficiently accurate. If we considered other published methods or procedures for the *J* integral assessment, it may be found that they are mostly concerned with concrete structural components subjected to specific loads with a partial employment of FEM calculation [29,30]. Finally, the EPRI method appeared to be highly qualified to provide sufficiently accurate *J* integral predictions. Moreover, the FC method (RCC-MRx Code) to which the GSM method is compared, is generally recognized as a standard used in the design of research reactors.

In principle, we can compare the FC and GSM *J–σ* curves among themselves, and then we can compare them with the EPRI *J–σ* curves. As seen in Figure 6, Figure 7, Figure 8, Figure 9, Figure 10, Figure 11, Figure 12, Figure 13, Figure 14, Figure 15, Figure 16 and Figure 17, the predicted FC and GSM curves beyond the SSY region are very steep, despite being relatively close to each other with the maximum differences in *J* values being around 20%. However, from a practical viewpoint, it is more advantageous to compare the FC and GSM curves on the basis of critical (fracture) gross section stress. For the highest value of the *J* integral in Figure 6, Figure 7, Figure 8, Figure 9, Figure 10, Figure 11, Figure 12, Figure 13, Figure 14, Figure 15, Figure 16 and Figure 17 (300 N/mm), the differences in gross section stresses as determined by the FC and GSM methods were 10–14 MPa for the relative crack length a/b = 1/8; 5–12 MPa for the relative crack length a/b = 1/4; 1–7 MPa for a/b = 1/2 and 1–5 MPa for a/b = 3/4. When we referred these differences to the corresponding gross section stresses, we found that the relative stress differences vary between 0.8% and 3.5%. From the diagrams presented in Figure 6, Figure 7, Figure 8, Figure 9, Figure 10, Figure 11, Figure 12, Figure 13, Figure 14, Figure 15, Figure 16 and Figure 17, it follows that FC and GSM predictions of *J* integral for specimens CCP, DECP and SECP were conservative for most of the configurations. The situations, when they are not conservative, are connected with small crack lengths (a/b = 1/8). The FC prediction is also not conservative for (i) X52 and X70 CCP plane stress specimens of a/b = 1/4, and (ii) X52 and X70 DECP plane stress specimens of a/b = 1/4. The GSM predictions were slightly more conservative than the FC predictions for all the cases investigated, with one exception being the X52 DECP plane stress specimens of a/b = 1/8. The biggest differences between the *J* integral predictions made using the FC and GSM methods, on the one hand, and those made using the EPRI method, on the other hand, were found to be for small cracks (a/b = 1/8) in both the X52 and X70 DECP specimens in plane stress as well as in most configurations of SECP specimens from both steels.

### 5.2. Cracked Pipes

As seen from the data in Table 5, the predicted magnitudes of the fracture pressure were conservative for both cracks. Those obtained by the GSM and FC method were close to each other, although the GSM predictions were slightly more conservative. The FEM prediction, although supposed to provide accurate results, appeared to predict conservative fracture pressures. The likely reason for this was the substitution of the real stress–strain dependence of steel with the Ramberg–Osgood approximation. When plotting these two curves on one diagram it could be seen that, from a certain point on the *σ–ε* curve onward, the Ramberg–Osgood stress became steadily greater than the actual stress, resulting in a greater strain energy density and thus in a higher magnitude of the *J* integral. The predicted fracture pressure for *J* = *J_cr_* then became naturally smaller than that found experimentally. As a matter of interest, Figure 21 and Figure 22 showed that the FEM predicted fracture pressures came close to the limit load pressures determined by Equation (41).

## 6. Conclusions

The main objective of this study was (i) to compare the *J* integral assessments as performed by the GSM and FC methods for the center-cracked panels (CCP), double-edge cracked panels (DECP), and single-edge cracked panels (SECP) made from steels X52 and X70, with predictions by the EPRI method, and (ii) to compare the *J* integral assessments as performed by the GSM and FC methods for a longitudinally cracked pipe produced from X70 steel, with the results obtained by finite element calculations and with the resulting burst pressures obtained experimentally. Both aspects of the main objective were achieved.

The results of *J* integral predictions for cracked panels showed that both methods provided very close predictions. In comparison with the EPRI results, these predictions were found to be conservative in most cases. The GSM predictions are slightly more conservative than the FC predictions for all the cases investigated, with one exception referring to X52 DECP plane stress specimens of a/b = 1/8.

The *J* integral predictions by the FC and GSM methods for the longitudinally cracked pipe appeared to be very close, the GSM prediction being slightly more conservative than the FC prediction. However, these predictions were widely conservative in comparison with the prediction by the FEM analysis. As far as the fracture toughness *J_cr_* = 439 N/mm for X70 steel is concerned, the corresponding magnitudes of the burst pressure for two longitudinal part-through cracks were: 7.16–7.38 MPa according to GSM and FC; 8.56–8.60 MPa according to FEM; and 9.55–9.86 MPa obtained experimentally. It follows from this that there exists a high degree of conservatism in predicting burst pressures of cracked pipes on the basis of the GSM and FC methods.

However, when operating high-pressure pipelines or cylindrical pressure vessels, the most important aspects that should be observed are the safety of the operation and the integrity of the pressure systems throughout their entire projected lifetime. It is for this reason that such methods of assessment of the life of pressure vessels and pipelines are preferred; so that they provide a conservative prediction of burst pressure across a wide range of possible stress states. With regard to thin-walled cylindrical pressure vessels and pipelines, the FC and GSM methods can be ranked among such methods. It has been proven here that the predictions of the *J* integral for thin-wall pressure pipelines using the FC and GSM methods is more conservative than those made using the FE method. On the other hand, the FE method provided lower magnitudes of fracture pressure than those found experimentally, i.e., it was conservative. It is very likely that the cause for this disagreement consists in substituting the real stress–strain dependence of the steel with the Ramberg–Osgood approximation.

Finally, it can be stated that the main contribution of this study is verification of the applicability of the newly developed GSM method for assessment of the *J* integral for cracked plates and pipes. Together with the FC method, it can be used for a conservative prediction of fracture parameters for cracked plates as well as for cylindrical pressure vessels and pipelines with relatively deep longitudinal part-through cracks.

## Figures and Tables

**Table 1:** The Ramberg–Osgood parameters and ultimate strengths of the steels used.

Material	Characteristics			
	α (1)	n (1)	σ_0_ (MPa)	R_m_ (MPa)
X52	2.40	6.25	313	493
X70	5.92	9.62	536	644

**Table 2:** Limit load parameters for the specimens used.

Specimen	C	
	Plane Stress	Plane Strain
CCP	1	2/√3
DECP	2/√3	0.91 + 0.36/(1 − a/b)
SECP	1.072 ψ	1.455 ψ

A note: parameter ψ is given by Equation (32).

**Table 3:** Some characteristics referring to the pipe segment.

Characteristics	Crack A	Crack B
Crack dimensions		
half-length, c (mm)	115	127
depth in fracture, af (mm)	7.1	6.7
Ramberg–Osgood parameters		
α/n/σ0 (MPa)	5.92/9.62/536	5.92/9.62/536
Flow stress		
σfs (MPa)	590	590
Fracture toughness		
Jcr = Jm (N/mm)	439	439
Fracture pressure		
pf (MPa)	9.55	9.86

**Table 4:** Geometric characteristics of the pipes with cracks, crack depth at fracture, and fracture pressure.

Quantity	*R_i_* (mm)	*t* (mm)	*c* (mm)	*a_f_* (mm)	*p_f_* (MPa)
Crack A	497.8	11.7	115	7.1	9.55
Crack B	497.8	11.7	127	6.7	9.86

**Table 5:** Comparison of predicted and experimental fracture pressures.

Quantity	*p_GS_* (MPa)	*p_FC_* (MPa)	*p_FEM_* (MPa)	*p_L_* (MPa)	*p_exp_* = *p_f_* (MPa)
Crack A	7.16	7.26	8.56	8.63	9.55
Crack B	7.26	7.38	8.60	8.61	9.86

## Data Availability

All data generated or analyzed during this study are included in this published article.

## Notation

The following symbols are used in this paper:

*a*	*=* crack length (depth);
*b*	*=* specimen width;
*c*	= half crack length;
*C*	*=* limit load parameter;
*D*	*=* outside diameter of a pipe;
*E*	*=* Young’s modulus;
*f*	*=* specific function of the ratio *a*/*b* for cracked specimens;
*J, J_cr_*	= *J* integral, critical *J* integral;
*J_e_*	= elastic component of *J* integral;
*J_m_*	= *J* integral corresponding to the maximum load in testing CT specimens;
*J_pl_*	= plastic component of *J* integral;
*k_t_*	= theoretical stress concentration factor;
*K_I_, K_I_^p^*	= stress intensity factor for mode I, provisional *K_I_*;
*n*	= Ramberg–Osgood exponent;
*p*	= internal pressure in a pipe;
*p_L_*	= limit internal pressure;
*p_Y_*	= yield internal pressure;
*P*	= acting load;
*P_0_*	= reference load;
*P_L_*	= limit load;
*R, R_i_*	= mean radius, internal radius of a pipe;
*R_m_*	= ultimate tensile strength;
*t*	= wall thickness of a pipe;
*w*	= strain energy density;
*α*	= Ramberg–Osgood constant;
*ε_o_*	= elastic strain at the yield stress;
*ε_e_*	= elastic strain at the reference stress;
*ε_ref_*	= reference strain;
*η, ξ*	= geometric parameters for a surface semi-elliptical crack in a pipe wall;
*σ_n_*	= net section stress or hoop stress in the ligament surrounding a longitudinal surface crack in a pipe wall;
*σ_o_*	= yield stress;
*σ_ref_*	= reference stress;
*σ_fs_*	= flow stress;
*σ_φ_*	= hoop stress;
(*ρ*, *θ*)	= polar coordinates of a point on the periphery of the notch root;
*φ*	= correction function for small plastic zone;
*ψ*	= geometric parameter for SECP specimens;
